# Structural and electronic transformation pathways in morphotropic BiFeO_3_

**DOI:** 10.1038/srep32347

**Published:** 2016-09-01

**Authors:** P. Sharma, Y. Heo, B.-K. Jang, Y. Y. Liu, J. Y. Li, C.-H. Yang, J. Seidel

**Affiliations:** 1 School of Materials Science and Engineering, UNSW Australia, Sydney NSW 2052, Australia; 2 Department of Physics, Korea Advanced Institute of Science and Technology, Daejeon 305-701, Republic of Korea; 3 School of Materials Science and Engineering, Xiangtan University, Xiangtan, Hunan 411105, China; 4 Department of Mechanical Engineering, University of Washington, Seattle, Washington 98195-2600, USA; 5 Institute for the NanoCentury, KAIST, Daejeon 305-701, Republic of Korea

## Abstract

Phase boundaries in multiferroics, in which (anti-)ferromagnetic, ferroelectric and ferroelastic order parameters coexist, enable manipulation of magnetism and electronic properties by external electric fields through switching of the polarization in the material. It has been shown that the strain-driven morphotropic phase boundaries in a single-phase multiferroic such as BiFeO_3_ (BFO) can exhibit distinct electronic conductivity. However, the control of ferroelectric and phase switching and its correlation with phase boundary conductivity in this material has been a significant challenge. Supported by a thermodynamic approach, here we report a concept to precisely control different switching pathways and the associated control of electronic conductivity in mixed phase BFO. This work demonstrates a critical step to control and use non-volatile strain-conductivity coupling at the nanoscale. Beyond this observation, it provides a framework for exploring a route to control multiple order parameters coupled to ferroelastic and ferroelectric order in multiferroic materials.

Sensing and actuation applications require usage of materials with ideally giant mechanical responses upon application of external stimuli. Over the years, various type of materials have been employed with the aim to address a wide variety of applications, examples of which include shape-memory alloys^1^,^2^, ferroelectrics^3^, and magnetostrictive materials^4^. Among the various alternatives, ferroelectrics, which are a subset of piezoelectric materials offer superior performance especially in high-frequency applications^5^. Moreover, their response can be enhanced greatly in the vicinity of a so called morphotropic phase boundary (MPB). At the MPB, piezoelectrics undergo structural phase transformation^6^,^7^, which typically gives rise to enhanced electromechanical coupling and large dielectric responses^8^ that can be exploited for applications ranging from electrostrictive actuators and sensors to energy harvesting. Traditional examples of piezoelectric morphotropic systems include composition-driven MPBs in ferroelectric perovskite oxides^3^,^9^,^10^ such as Pb(Zr_1−x_Ti_x_)O_3_, (PbMg_1/3_Nb_2/3_O_3_)_1−x_–(PbTiO_3_)_x_, and hydrostatic pressure driven MPBs in pure PbTiO_3_ single crystals^6^,^11^. Recently, a fundamentally new approach comprising of a strain-driven MPB in multiferroic BiFeO_3_ (BFO) thin films, which emulate the performance of traditional Pb-based morphotropic systems was successfully demonstrated^12^. This approach introduces a high performance recyclable lead-free alternative for potential high-frequency electromechanical applications. Via thin-film strain engineering^12^, the ground state of BFO can be tuned from a rhombohedral phase in the bulk to a novel tetragonal-like (T-like) phase with monoclinic distortion. To assist with strain relaxation and release the elastic energy, another rhombohedral-like (R-like) monoclinic phase starts to emerge with increasing film thickness, and forms self-organized corrugated nanoscale regions in coexistence with the T-like phase^12^,^13^,^14^,^15^. At these corrugated nanoscale regions in which both phases coexist, the phase boundaries between the T- and R-like phases represent morphotropic phase boundaries across which crystal structure evolves continuously from one structural phase to the other. The strain-driven MPBs in BFO are physical boundaries typically a few unit cells wide (2…10 unit cells), which spatially separate R- and T-like phases and across which an enormous lattice mismatch of up to approximately 14% can be accommodated^16^. At this MPB, giant electromechanical responses have been observed similar to the traditional Pb-based morphotropic systems^16^. These arise mainly because of the drastic changes in the crystal structure, and the associated strain gradients at the phase boundaries^17^,^18^. In addition to the enhanced piezoelectric coupling, other novel functionalities have been shown to emerge at these strain-driven MPBs due to accompanying changes in spin and electronic structure, e.g. enhanced spontaneous magnetic moments^19^, and electrical conduction^20^,^21^,^22^. There are various control mechanisms such as strain, thickness, chemical doping, external electric field, and mechanical stimulus available^16^,^23^,^24^,^25^,^26^,^27^, via which these structural phase boundaries can be manipulated in a non-volatile manner to tune competing and interdependent physical properties connected with the associated structural-phase transformations at the MPB^28^,^29^,^30^,^31^,^32^. However, a precise nanoscale control along with a detailed understanding of the structural and electronic transformation pathways mediating giant electromechanical coupling in the strain engineered morphotropic BFO thin films is missing. Besides, interconnection of these nanoscale phase transformations with electrical conduction properties is yet another aspect that remains unexplored. In the present work, we address these significant challenges and provide a direct nanoscale insight into the enhanced mechanical responses including coupled electrical transport behavior.

In this study, structural and electronic phase transformations have been visualized directly at the nanoscale via employing the ‘*spectroscopic static-piezoresponse*’ approach, which elucidates the origin of giant electromechanical coupling in the morphotropic BFO thin films. Several structural and electronic transformation pathways have been experimentally realized using external bias as a control parameter applied through the conductive tip of an atomic force microscope (AFM). This approach provides a direct quantification of the electrically recoverable strain during various transformation pathways, which are well supported theoretically by a thermodynamic model. Finally, a strong correlation between the observed phase transformations mediating enhanced electromechanical response and electrical characteristics of the morphotropic BFO thin films has been observed, revealing clear contributions to the conductivity from structural and polarization transformations.

## Methods

For this study, La(5%)-substituted BFO thin films (Bi_0.95_La_0.05_FeO_3_, BLFO) were grown with a conducting buffer layer of Pr_0.5_Ca_0.5_MnO_3_ on (001) LaAlO_3_ substrates using pulsed laser deposition with a KrF (krypton fluoride laser) excimer laser (λ = 248 nm). We used a BLFO ceramic target (Bi_1.05_La_0.05_FeO_3_) with Bi at 10% excess to prevent bismuth loss during pulsed laser deposition growths. The laser energy was 110 mJ, and the frequency was 10 Hz. During growth, the substrate temperature was kept at 650 °C and oxygen partial pressure was set to 100 mTorr. After growth was completed, the samples were cooled to room temperature at a rate of 10 °C per minute with an oxygen environment of 500 Torr. The film thickness of the BLFO layer was approximately 60 nm^22^,^27^. For comparison, similarly thick pure BFO thin films were also prepared on (001) LaAlO_3_ substrates with a conducting buffer layer of Pr_0.5_Ca_0.5_MnO_3_ (PCMO). The scanning probe microscopy (SPM) measurements were performed using commercial AFM systems (Cypher-Asylum Research and AIST-NT Smart SPM 1000) at room temperature under ambient conditions. Ti-Pt coated (force constant, k = 8–40 N/m), and diamond-coated (k = 28–91 N/m) Si cantilevers were used for the SPM measurements. Dynamic piezoresponse measurements were performed using an ac imaging voltage with frequencies in the range of 350–800 kHz, and with amplitude of 0.6 V (peak-to-peak). The measurements of local electrical current were implemented using ORCA module (Asylum Research). In our AFM set-up, currents in the range of few pA to 20 nA (upper compliance limit in our set-up) can be measured accurately.

## Results and Discussion

A typical topographic image of the 60 nm 5% La-doped BFO (BLFO) sample shows mixed-phase areas embedded in the parent T-like phase matrix (Figure S1, supplementary information, section I) and is consistent with the previously reported morphological and structural results^22^,^27^. The T-like phase matrix also referred to as the M_II_ phase according to the convention developed in ref. 33 reveals atomically flat terraces corresponding to single unit-cell high steps of approximately 4 Å (see supplementary information, section I). While, the mixed-phase areas display a typical ordered corrugated stripe patterns consisting of competing T-, and R-like phases. The R-like phase in the mixed-phase area (referred to as M_I_ phase^33^) appears dark in the topography image due its smaller *c*-axis lattice parameter compared to the T-like phase. The T-like phase in the mixed-phase area, also referred to as M_II, tilt_ phase^33^ has the same *c*-axis lattice parameter as the parent phase (M_II_ phase) outside, but tilted away from the sample normal (i.e. [001]) by approx. 1.5°^33^. In the remainder of this article, we do not distinguish between M_II, tilt_ and M_II_ phase, and simply refer to them as the T-like phase. Further, detailed structural information about these samples can be found elsewhere^22^,^27^. The structural phase variants of the morphotropic BLFO thin films were responsive to external electrical or the mechanical stimulus, which allows their manipulation in a rewritable fashion^22^,^27^,^29^.

Previously, structural and polarization transformations in the morphotropic BFO thin films have been inferred via simultaneously acquired piezoresponse (piezoresponse force microscopy: PFM) and topographic images after application of an external bias^12^,^33^, and external-field caused variation of electromechanical response as observed in the spectroscopic dynamic PFM hysteresis loops^17^,^28^. Probing using SPM-based imaging approaches after application of incrementally changing local electrical bias is nonetheless valuable, but is limited in kinetic detail as the data is acquired after application of electrical bias. Besides, SPM-based imaging approaches require considerable amount of time (in the range of several hours) to obtain the full field-induced hysteretic response^12^,^33^. Investigations using spectroscopic methods such as local dynamic piezoresponse spectroscopy improve temporal resolution and shortens data acquisition span, but requires calibration of the AFM-cantilevers and inverse optical lever sensitivity of the AFM detection scheme to quantify the observed nanoscale electromechanical behavior.

**Figure 1:**
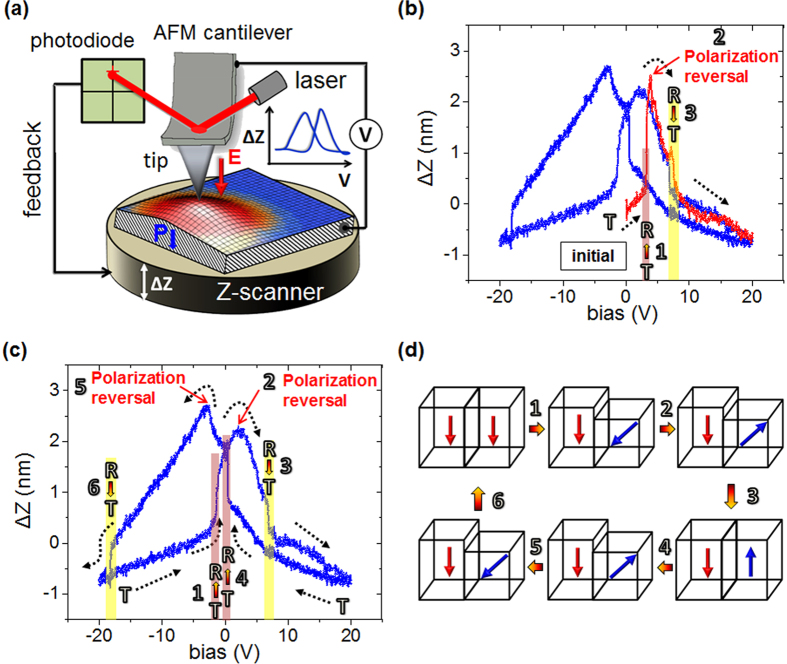
Giant electromechanical response in morphotropic La-doped BiFeO_3_ thin films. (**a**) A schematic of the experimental geometry. (**b**) Spectroscopic local surface displacement as a function of applied external DC bias. (**c**) Same data as in (**b**), but without the initial quarter cycle. (**d**) A schematic illustration of the evolution of nanoscale phase transformations mediating the giant electromechanical response in BFO thin films.

In this article, we have employed a simple yet powerful spectroscopic approach known as static-piezoresponse approach^34^,^35^, which overcome the limitations discussed with the previous approaches. The spectroscopic static-piezoresponse approach quantitatively elucidates the detailed external field-induced nanoscale electromechanical behavior including phase transformations, transformation pathways and the coupled electrical transport behavior in the multiferroic morphotropic BFO thin films. This spectroscopic approach directly visualizes the kinetic details of nanoscale phase transformations and quantifies the observed field-induced response without the need for calibration of the cantilevers and AFM optical detection scheme. This approach involves monitoring the surface displacement of the electro-active ferroelectric film as a slowly varying DC bias is supplied between the conductive nanoscale tip and bottom electrode of the sample in the contact mode. Due to intrinsic piezoelectric coupling, the surface of the electro-active ferroelectric film deforms on application of an external electrical bias. The surface of the ferroelectric film contracts if the applied external field direction is opposite to the polarization direction or expands if the external field is in the same direction as polarization. The external-field caused nanoscale surface deformation of the ferroelectric film sets *z*-piezo (*ΔZ*) of the AFM in motion via a feedback mechanism to maintain a constant loading force between tip and sample. Driven by feedback-circuitry, *z*-piezo (*ΔZ*) of the AFM brings the sample either towards or away from the AFM-tip depending upon whether the surface of the sample undergoes contraction or expansion respectively under an applied external bias. As a result, nanoscale surface deformation as a function of applied external electrical bias can be monitored directly by recording the motion of *z*-piezo (*ΔZ*) of the AFM (Fig. 1(a)). Following this recipe, one can visualize nanoscale surface deformations including structural and electronic phase transformations (further details in supplementary information, section II). The structural transformations can be deciphered unambiguously especially in the morphotropic systems such as BFO because the transitions between well-established competing structural phases involve significant change in the out-of-plane lattice constant and consequently surface morphology^12^,^13^,^14^,^33^. Therefore, the structural transitions manifest as an abrupt jump in the acquired response, while electronic transformations associated with flipping in the direction of the ferroelectric polarization are marked by reversal in the sign of the acquired response or strain^16^,^33^.

Figure 1(b) displays a typical nanoscale surface displacement versus DC bias hysteretic curve as observed on as-grown T-like phase region of the BLFO film (i.e., on area outside the mixed-phase, see supplementary information, section I). In these measurements unless stated otherwise, the bias is supplied to the bottom electrode, and the tip is held at ground. Firstly, the observed surface displacement versus bias curve (Fig. 1(b,c)) reveals a distinct butterfly-shaped non-linear hysteretic loop resembling that observed in classical ferroelectric materials such as Pb(Zr, Ti)O_3_, and PbTiO_3_ (see Figure S2, supplementary information, section III). Further, it reveals a giant electric field-induced reversible strain of about 6% (i.e., strain, *ε* = *ΔZ*_max_*/L*, *L* is film thickness, which is 60 nm), which is mediated by nanoscale structural phase transformations, and the polarization reversal effects. In fact, a closer look at the observed bias-induced surface displacement data elucidates the nanoscale dynamic evolution of structural and electronic phase transformations in the morphotropic BLFO thin films. This nanoscale phase evolution is sketched schematically in Fig. 1(d).

Initially, *ΔZ* increases linearly with the bias, because the applied external field is directed opposite to the polarization direction (i.e., in the as-grown state, the polarization is pointing downwards towards the bottom electrode, see supplementary information, section IV) of the T-like phase of the BLFO film resulting in surface contraction, and thus a positive upward motion (*ΔZ*) of *z*-piezo of the AFM to keep the tip-induced loading force constant. With increasing bias, a distinct abrupt increase (of approx. 1 nm) in the *ΔZ* occurs at a DC bias of approx. +3.5 V, which marks the onset of the structural-phase transformation from the initial T-like phase to the R-like phase (due to its smaller *c*-axis lattice parameter)^27^,^33^, designated as “event 1” in Fig. 1(b,d). Based on earlier published reports^22^,^30^,^33^, the polarization of the R-like phase is tilted away from the surface normal compared to the nearly vertical orientation in the T-like phase. Therefore, the structural transformation as observed (“event #1”) in Fig. 1(b) is likely accompanied by polarization rotation away from the surface normal. Though, the polarization of the newly formed R-like phase is still pointing downwards. After structural-phase transformation, the *ΔZ* continues to increase with the applied bias due to increasing surface contraction until it reaches “point #2” (Fig. 1(b)). At point #2, the polarization of the newly formed R-like phase flips from downward to the upward direction (DC bias approx. +4 V), and results in the reversal of sign of strain, i.e., from there on the *ΔZ* decreases with increasing bias. This in fact matches very well with the observed coercive bias values as measured using spectroscopic dynamic PFM hysteresis loops on the same sample (supplementary information, section V). Similar measurements on a standard ferroelectric sample such as PbTiO_3_ which do not involve the complexity of the structural-phase transformations further validate this scenario, whereby out-of-plane polarization reorientation is associated with the reversal in the sign of strain or surface displacement (see supplementary information, Fig. S2).

Therefore, after polarization reversal (R to −R phase), the polarization of the R-like phase points upwards, and *ΔZ* decreases with the increasing bias due to increasing surface expansion of the BLFO film. At a bias of approx. +8 V (“event #3”, Fig. 1(b)), the BLFO film undergoes a reverse structural phase transformation, i.e. from R-like phase to the T-like phase, which is highlighted again by a sharp change in the surface height. The transformation to the T-like phase represents the as-grown T-like phase however, with reversed orientation of the polar vector (i.e. −T phase). On increase of the bias after reverse structural transformation (i.e. −T phase), *ΔZ* decreases with increasing bias in a nearly linear manner.

On decrease of the bias from +20 V to 0 V, the *ΔZ* increases accompanied by an abrupt transition at a bias of about +0.5 V (“event #4”, Fig. 1(b,c)), indicating the onset of structural phase transformation from the T- to the R-like phase (i.e., from −T to −R phase). On further decrease of the bias in the negative direction (0 to −20 V), the sign of the strain or surface displacement again changes direction indicating polarization reorientation (i.e., from −R to R phase) as marked by “event #5”. After polarization reversal, the *ΔZ* decreases with the bias (in the negative direction), and at a bias of approx. −18 V, another structural transformation takes place (“event #6”), in which the R-like phase transforms back to the initial/as-grown T-like phase with polarization pointing towards the bottom interface. Afterwards, on retrace of bias from −20 to +20 V, same sequence of transitions marked earlier by “events #1–3” (Fig. 1(b,c)) can be uniquely identified.

In this simple, yet effective approach, we not only distinctly identify all nanoscale events #1 to 6 as sketched schematically in Fig. 1(d), but also visualize their sequence and characterize the external biases/fields at which respective effects such as structural transformations, phase boundary motion, and polarization reorientation are triggered. In particular, the structural phase transformations between well-established T- and R-like phases of the BFO are uniquely marked by an abrupt jump (i.e., event #1, 3, 4, and 6 in Fig. 1(b,c)) of approximately 1 nm in the surface displacement^24^,^27^, whereas the polarization flipping is accompanied by the reversal in the direction of surface displacement as highlighted by event #2, and 5 in Fig. 1(b,c)^34^,^35^. Clearly, the giant nanoscale electromechanical response in morphotropic BFO thin films is mediated by reversible structural phase transformations (two T- to R-like phase transformations, and two reverse R- to T-like phase transformations), motion of the morphotropic phase boundaries, and rotation of the ferroelectric polarization.

A few general features of the observed nanoscale phase transformations as inferred from the experimental data are summarized as follows. First, it is worth noting that the structural-phase transformations of both types, i.e. from T- to the R-like phase, and from R- to the T-like phase can be accomplished by applying bias of the same sign (e.g., in Fig. 1(c): for negative bias- events #1 and 6, and for positive bias- events #3 and 4) depending upon its magnitude and history of the previously applied bias^33^. Further, it is interesting to note that the T- to R-like structural phase transformation occurs at low biases in contrast to the reverse transformation. Also, after the initial first quarter cycle (red curve in Fig. 1(b)) in which the bias was ramped to a relatively high value (i.e. 20 V), the subsequent T- to R-like structural phase transformations occur at bias of around 0 V. This suggests that the as-grown/initial T-like phase after being subjected to a relatively high bias is no longer stable in the absence of bias, and indicates a strong tendency towards the formation of nanoscale mixed-phase region (i.e., R-like phase). This is also clear from the decrease in the threshold bias for the T- to R-like structural phase transformation from +3.5 V to nearly 0 V, after the initial first quarter cycle. Moreover, the acquired hysteretic response as shown in Fig. 1(b,c) is not symmetric. The asymmetry likely arises from charge injection into the film under applied relatively high biases (V_DC_ > 15 V)^36^,^37^. The injected charge dynamics possibly affects not only the threshold biases for subsequent structural phase transformations, but also the acquired response. This conclusion is further reinforced by the observation of nearly symmetric hysteretic response in the applied bias range of V_DC_ < 15 V (see supplementary information, section VI).

**Figure 2:**
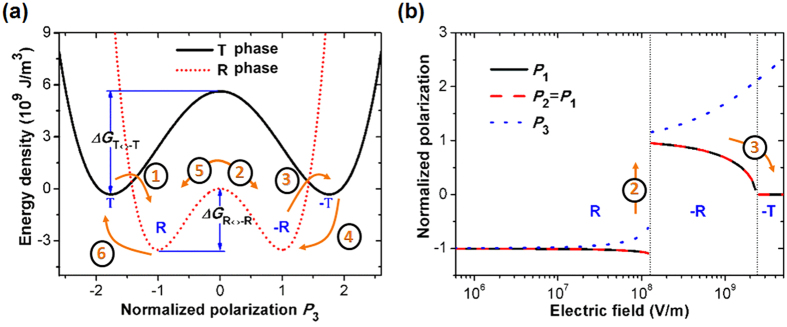
Calculated free energy density profiles for the respective structural phase variants of the BFO film. (**a**) Energy barrier between T and −T phase, as well as R and −R phase under zero electric field. (**b**) The evolution of R phase under [001] electric field. In (**a**), numbers 1–6 and arrows indicate likely sequence of phase transformations (as experimentally observed in Fig. 1) under an applied external electric-field loading cycle.

The experimentally observed sequence of structural and electronic transformations in the above described pathway is well explained on the basis of a thermodynamic model (see supplementary information for details, section VII). Under the proposed model, zero-field free energy density profiles with respect to polarization (*P*_3_) for tetragonal and rhombohedral-like structural phases of the BFO films are evaluated (Fig. 2). To simplify theoretical calculations, we consider *P*_1_ = *P*_2_ = 0, *P*_3_ ≠ 0 for the T-like phase, while *P*_1_ = *P*_2_ = *P*_3_ (this is the case for BFO in bulk) for R-like phase or simply R phase. In these calculations, polarization is normalized with respect to *P*_3_ of the R-phase at the minimum of the free energy density (i.e. at the energy well). It is found that the energy barrier between T and −T (i.e. T phase with reversed polarization) phase is higher than that of energy barrier between R and −R phase (*ΔG*_T↔−T_ > *ΔG*_R↔−R_). This observation suggests that the polarization switching between T and −T phase is more difficult to accomplish than between R and −R phase. In the strain-driven MPB, where both phases coexist, the T → −T phase transition is mediated by the R-like phase (Fig. 1b,c)^12^,^28^. Therefore, with increasing electric field, R-like phase undergoes polarization reversal transforming to the −R-like phase before its transition subsequently to the −T-like phase, and agrees with expected sequence of transitions based upon calculated free energy density profiles for the respective structural phase variants. Similar as the quarter cycle (starting from 0 V to positive biases) in Fig. 1(c), we examine polarization evolution under [001] electric field (Fig. 2(b)), which clearly shows R → −R phase transformation before −R → −T transformation with increasing electric field, and agrees well with the observed experimental data. Also from the calculations (Fig. 2(a)), the energy well of the R phase is lower than that of T phase, suggesting that the T → R (or −T → −R) phase transformation is likely to occur, even when the electric field is decreasing, and explains strong tendency towards formation of nanoscale mixed-phase regions. Thus, theoretical calculations suggest a sequence of transformations, i.e., (T → R → −R → −T) with increasing electric field, and (−T → −R → R → T) with decreasing field/increasing field in opposite direction, that can be realized under an electric-field loading cycle, which is consistent with the experimental observations (Fig. 1(b,c)). These theoretical calculations were performed for isolated structural phases of the BFO film, though at MPB, where both phase variants coexist, a composite free energy density profile is suggested. The composite free energy density profile in the first order approximation is the sum of independent free energy density profiles as calculated for the respective structural phases at the MPB. This composite profile is expected to have several local minima’s, such that the energy barrier between them governs the dynamic evolution of structural and electronic phases of the BFO film.

**Figure 3:**
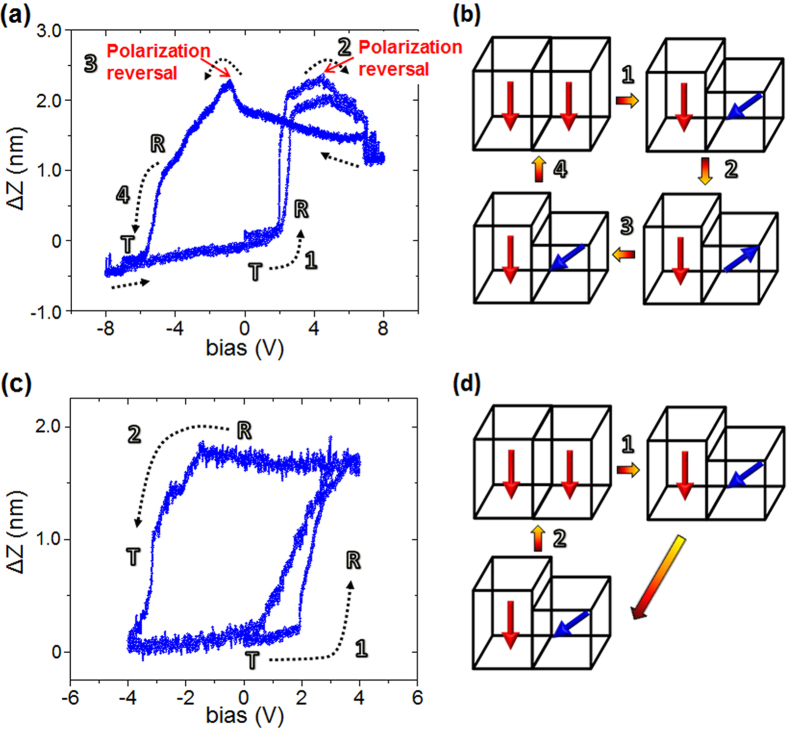
Bias-dependent pathways for the phase transformations in morphotropic La-doped BiFeO_3_ thin films. (**a**) Spectroscopic local surface displacement versus electrical DC bias in the medium cycled bias range of ± 8 V. (**b**) A schematic illustration of the evolution of nanoscale phase transformations as elucidated from the acquired static-piezoresponse in (**a**). (**c**) Spectroscopic local surface displacement as a function of applied external DC bias as observed in the relatively low applied bias range of ±4 V. (**d**) A schematic illustration of the nanoscale phase transformations corresponding to the acquired static-piezoresponse in (**c**).

With that in mind the question arises whether it is possible to achieve different sequences of phase transformations depending upon the magnitude of the applied electrical bias. The answer is yes, and this is confirmed by the measurements shown in Fig. 3(a,b). In this case, the maximum applied bias was limited to ±8 V, and clearly a different pathway of the nanoscale phase transformations is observed. In this case, as before, the measurements were performed at a fixed location on the initial T-like phase. With increase in the bias from 0 to +8 V, two events take place in succession at the nanoscale. Initially, the *ΔZ* increases smoothly with the applied electrical bias followed by an abrupt jump, which marks the occurrence of structural phase transformation from the initial T- to the R-like phase (at a bias of approx. +2 V, “event 1”, Fig. 3(a)). Afterwards, the polarization of the R-like phase flips from downward to the upward direction (“event 2”, at a DC bias of approx. +4 V, Fig. 3(a)), and results in the reversal of sign of strain, i.e. *ΔZ* decreases with increasing bias. As bias is swept in the reverse direction (+8 V to −8 V), the *ΔZ* increases with decreasing bias until (event #3, at a bias of approx. −1.5 V, Fig. 3(a)) polarization of the R-like phase reorients back to the downward direction. Thereafter, the *ΔZ* decreases with the decreasing bias, and is accompanied by an inverse structural phase transformation (from R-like phase) back to the initial T-like phase. Further, with retrace of electrical bias from −8 to +8 V, the transitions identified (as “events #1–2”) earlier were reproduced.

The sequence of transitions as visualized in the surface displacement versus bias data (Fig. 3(a)) is schematically sketched in Fig. 3(b). Evidently, by restricting the magnitude of the applied DC bias, one can select a distinct pathway (pathway-II, Fig. 3(a,b)) for the nanoscale phase transformations. In pathway-II (Fig. 3(a,b)), for the bias of same sign, the applied DC bias magnitude is below the local bias threshold needed for the reverse transformation (for e.g., “event #3” for positive bias, and “event #6” for the negative bias in Fig. 1(b,c)), which changes the order/sequence of the transitions visualized over a relatively large bias range. In pathway II, the T- to R-like phase transformation occurs at a low bias magnitude in contrast to the inverse transformation, and is in agreement with the observations made for the pathway-I (Fig. 1). However, in this particular scenario (Fig. 3(a,b)), the competing phases can be reversibly transformed in to one another by applying biases of opposite sign, and of suitable magnitude, and shows distinct behavior from pathway-I in which bias of same sign depending upon its magnitude and prehistory can be selectively used to accomplish both type of (i.e., T- to R-like phase and R- to T-like phase) structural-phase transformations.

By further restricting the magnitude of the applied electrical bias, one can select a still different pathway (from the two previous, i.e., pathway-III) for the phase transformations, as shown in Fig. 3(c), and schematically sketched in Fig. 3(d). In this case, the applied DC bias is below the local polarization switching threshold. Thus, the strain/surface displacement hysteresis curve (Fig. 3(c)) is mediated only by the structural phase transformations without reversal of the out-of-plane component of the polarization. As is clear (Fig. 3(c,d)), the initial T-like phase transforms to the R-like phase, which subsequently transforms back to the initial T-like phase.

Figures (1, 2, 3), thus demonstrate that the bias magnitude is an effective control parameter to tune/select a particular sequence/order of nanoscale phase transformations including structural transitions, and polarization rotation/reversal events. At a relatively large applied bias range of ±20 V, a nanoscale strain of approx. 6% is accompanied by four structural-phase transformations (two R- to T-like, and two T- to R-like phase transformations), and two polarization reversal events. At a medium cycled bias range of ±8 V, a maximum nanoscale strain of about 4% is associated with two structural phase (one each R- to T-like, and T- to R-like phase transformation) transformations, and two polarization reversal events. However, at the low applied bias range of ±4 V, only structural-phase transformations (one each R- to T-like, and T- to R-like phase transformation) take place resulting in a maximum electrically reversible strain of about 3%. In all these measurements, nanoscale phase transformations were not only visualized, but the sign and magnitude of external biases/fields needed was also established. The threshold biases at which forward and reverse structural transformations take place were controlled in a dial-in manner depending upon the applied external bias range selecting a particular pathway. These local quantitative strain measurements distinctly illustrate the key role of reversible structural transitions, nucleation/movement of morphotropic phase boundaries mediating structural transitions, and polarization transformations resulting in giant nanoscale electromechanical response of the strained morphotropic BFO thin films.

It is noted that the small differences of 1–2 V in the threshold biases for the structural transformations (for e.g., among different pathways from the initial T- to the R-like phase) can arise from spatial inhomogeneities. Previously^30^, it has been shown that there are eight directions along which the R-like phase stripes can arise making an angle of approximately ±10° with respect to the neighboring in-plane 〈100〉 crystallographic directions. Thus, the orientation of the bias-induced nucleated R-like phase stripes during acquisition of the spectroscopic static piezoresponse is governed by mechanical compatibility conditions such as local strain environment and orientation of the surrounding stripes, which enclose the point where the bias is being applied^27^. Hence, spatial inhomogeneities can result in slight variations of threshold biases for the structural transformations. For pathway-I besides spatial inhomogeneities (as noted before), strong charge injection can occur during initial ramping of the bias to relatively high values (V_DC_ > 15 V) that can affect the threshold biases for the subsequent structural phase transformations (for e.g., event#6 in Fig. 1(c)).

**Figure 4:**
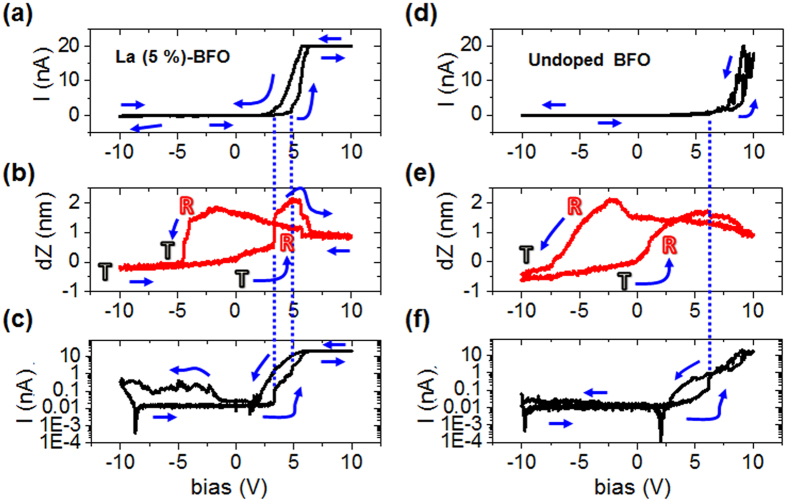
Coupling between phase transformations, and electrical characteristics. For the La-doped BFO sample (**a**,**b**), simultaneous recording of electrical current (**a**), and surface displacement (**b**), as a function of applied external DC bias. (**c**) Plot of electrical current versus DC bias as shown in (**a**) in the semi-logarithmic scale. For the undoped BFO sample (**d**,**e**), simultaneous recording of electrical current (**d**), and surface displacement (**e**), as a function of applied external DC bias. (**f**) Plot of electrical current versus DC bias as shown in (**d**) in the semi-logarithmic scale.

Further, we have investigated the interconnection of nanoscale phase transformations, and electrical characteristics of the morphotropic La-doped BFO thin films. For this purpose simultaneous measurements of surface displacement and electrical current were performed as a function of applied external electrical DC bias. The combined nanoscale mechanical and transport measurements were performed (in the medium applied external DC bias range, i.e., ± 10 V) on an initial T-like phase with results being shown in Fig. 4(a–c). First, the plot of the surface displacement versus DC bias (Fig. 4(b)) resembles the data presented in Fig. 3(a). The acquired response (Fig. 4(b)) shows an electrically recoverable strain of approx. 3.3% and is mediated by two structural-phase transformations, i.e., T- to R-like phase transformation at a bias of approx. +3 V, and the reverse R- to T-like phase transformation at a bias of approx. −4 V. Besides structural transformations, the polar vector undergoes reversal of its out-of-plane component followed by transition back to the initial state. The simultaneous nanoscale electrical measurements (Fig. 4(a)) on the other hand reveal a hysteretic Schottky diode-like behavior, which is consistent with previously reported electrical measurement results^22^. The hysteretic nature of the transport characteristics in that case was attributed to slow defect/ionic motion contribution to the electronic conduction, whereas the Schottky-like behavior arises from the work function difference between the tip and sample^20^,^22^. On closer inspection, the electrical conductivity exhibits a “sharp increase” (Fig. 4(c), from the I-V plot in the semi-logarithmic scale), at the structural phase transformation from the initial T- to the R-like phase. This nanoscale structural transformation results in the creation of physical morphotropic phase boundaries due to the presence of T-, and R-like phase polymorphs (Fig. 4(a–c)), which eventually lead to the enhanced electrical conductivity. This is consistent with the previously observed enhanced electrical conductivity at the isosymmetric phase boundaries in doped BFO, which was attributed to a variety of factors including interplay of local strain environment, octahedral tilts^20^, and bound charges arising because of polarization discontinuity^22^. The simultaneous fast “step-like” increase in the electrical conductivity on creation of isosymmetric phase boundaries point to the electronic nature of the electrical conduction at these structural phase boundaries. Furthermore, there is another step-like increase in the electrical conductivity on reversal of the polarization, which is likely a combination of the following mechanisms, i.e. polarization-controlled electrical transport^35^,^38^,^39^, and the ferroelectric domain wall conductivity^40^,^41^,^42^,^43^.

During the reverse structural phase transformation (from R- to the T-like phase), and polarization reorientation back to the initial state, no significant changes were observed in the electrical conductivity. This is likely due to the Schottky-nature of the electrical transport, and also due to the fact that the pure T-like phase (due to reverse transformation from mixed-phase to the initial T–like phase) in the absence of structural phase boundaries displays low electrical conductivity, i.e., high resistance^20^,^22^,^27^.

Furthermore, similar simultaneous measurements were performed on an undoped BFO sample (thickness approx. 60 nm) with results being shown in Fig. 4(d–f). As can be seen, the nanomechanical, and electrical behavior is similar to that observed in the La-doped BFO sample. However, there are few important noteworthy differences. In the undoped case, there is no sharp increase in the electrical conductivity at the T- to R-like structural phase transformation as is the case for the La-doped BFO sample. This behavior is rather consistent with the previous reports in which the enhanced conductivity at the phase boundaries was observed only for the doped (for e.g. La, and Ca-doped) BFO samples^20^,^22^. On further increase of the bias, similar to the La-doped BFO sample, there is a sharp increase in the electrical conductivity on reversal of the polarization. Additionally, for the undoped case it is important to point out that the hysteresis in the electrical data is significantly less (especially for negative biases), and thus is in agreement with observations made earlier about the defect/ionic motion contributing to the hysteretic electrical behavior of the doped samples.

**Figure 5:**
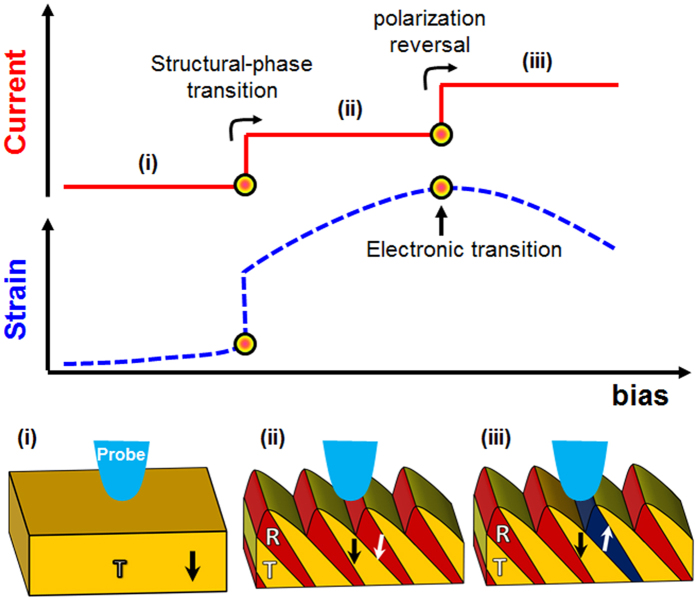
Non-volatile strain-conductivity coupling at the nanoscale. A schematic illustration of the interconnection between nanoscale electrical transport, and phase transformations in the La-doped BFO sample.

The simultaneous strain and transport measurements clearly demonstrate non-volatile strain-conductivity coupling at the nanoscale and provide a viable route to exploit this phenomenon at the local level. By tuning the applied bias range and thereby selecting a particular transformation pathway, a precise non-volatile control of the conductivity can be accomplished depending upon the bias-induced structural and electronic phase transformations mediating giant piezoelectricity of the morphotropic BFO thin films. In particular for the doped BFO thin films, multilevel resistance states can be realized by modulation of the underlying structural phase, and polarization as shown schematically in Fig. 5.

In summary, a spectroscopic static-piezoresponse approach was employed to directly and quantitatively elucidate the nanoscale origin of the giant electromechanical response coupled to electronic properties in morphotropic BFO thin films, which involve electrically reversible structural phase transformations, morphotropic phase boundary motion and electronic transformations. Under this approach, nanoscale phase transformations were not only visualized, but sign and magnitude of external biases at which the respective events are triggered were also established. Using applied external electrical bias as a control parameter, several selective and well-controllable pathways for structural and electronic transformations were experimentally demonstrated which were supported theoretically by a model based upon a thermodynamic approach. Furthermore, simultaneous monitoring of phase transformations and electrical characteristics established non-volatile strain-conductivity coupling at the nanoscale and decoded contributions of the external-field driven reversible structural and electronic polarization phase transformations. Our findings thus demonstrate nanoscale control of ferroelectric and phase switching pathways and their interconnection with the morphotropic phase boundary conductivity. These results provide novel insights into the superb electromechanical property and electrical conduction at the phase boundaries in morphotropic BFO thin films, and point to new avenues in the design and implementation of one-dimensional rewritable multifunctional nanostructures.

## Additional Information

**How to cite this article**: Sharma, P. *et al*. Structural and electronic transformation pathways in morphotropic BiFeO_3_. *Sci. Rep.* **6**, 32347; doi: 10.1038/srep32347 (2016).

## Supplementary Material

Supplementary Information
