# Supplementary material for: Structural and electronic transformation pathways in morphotropic BiFeO3
Source: Sci Rep. 2016 Sep 1;6:32347. doi: 10.1038/srep32347 (PMC5007483; doi:10.1038/srep32347)
Supplement: Supplementary Information [file srep32347-s1.doc]

**Supplementary Information**

**Structural and electronic transformation pathways in morphotropic BiFeO_3_**

**P. Sharma,^1*^ Y. Heo,^1^ B.-K. Jang,^2^ Y. Y. Liu,^3^ J. Y. Li,^4^ C.-H. Yang^2,5^ and J. Seidel^1**^**

^1^School of Materials Science and Engineering, UNSW Australia, Sydney NSW 2052, Australia

^2^Department of Physics, Korea Advanced Institute of Science and Technology, Daejeon 305-701, Republic of Korea

^3^School of Materials Science and Engineering, and Key Laboratory of Low Dimensional Materials

& Application Technology of Ministry of Education, Xiangtan University, Xiangtan, Hunan 411105, China

^4^Department of Mechanical Engineering, University of Washington, Seattle, Washington 98195-2600, USA

^5^Institute for the NanoCentury, KAIST, Daejeon 305-701, Republic of Korea

Email: *[pankaj.sharma@unsw.edu.au](mailto:pankaj.sharma@unsw.edu.au), [**jan.seidel@unsw.edu.au](mailto:**jan.seidel@unsw.edu.au)

1. **Morphology of the La-doped BFO film**

Figure S1 shows a typical morphology image of the 60 nm La (5%) doped BFO film. As can be seen (Figs. S1(a,c)), the corrugated nanoscale regions corresponds to the mixed-phase areas comprising of M_I_ and M_II,tilt_ phases (i.e. R- and T-like phases respectively) identified and named according to the convention developed in Ref [[[1]](#endnote-2)]. The area outside the mixed-phase regions is referred to the M_II_ phase (parent T-phase matrix), and shows single unit-cell high atomically flat terraces (Figs. S1(a,b)).


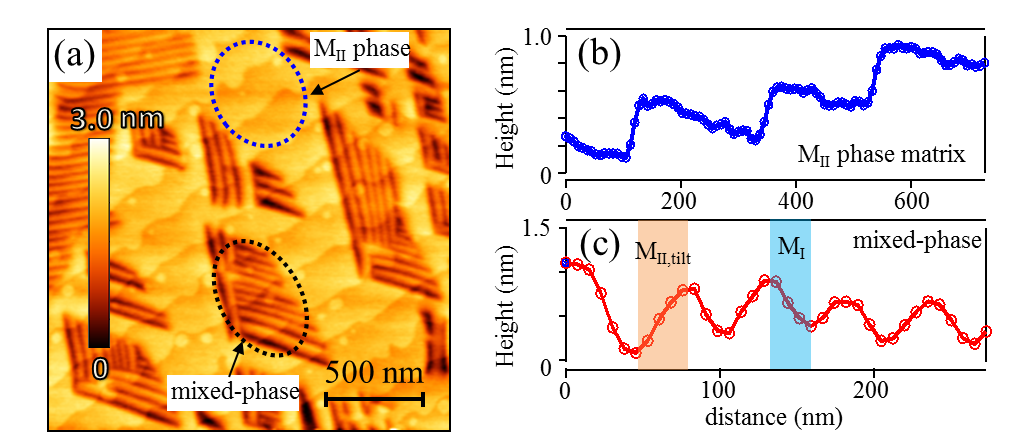


**Figure S1.** (a) Topography of the La-doped BFO sample. (b,c) Cross-section analyses along the M_II_ (parent T-like phase) phase (b), and the mixed phase region (c), as shown in the topographic image. In (c), blue-shaded region corresponds to M_I_ phase (i.e. R-like phase in the mixed-phase region), whereas the red-shaded region corresponds to M_II,tilt_ (i.e. T-like phase in the mixed-phase region) phase.

1. **Static-piezoresponse approach**

Under this approach, the position of the conductive AFM-tip is fixed at a predetermined location on the surface of the sample in contact mode (exerting a nominally low loading force of approx. 50 nN). Thereafter, a slowly varying (typical time period in the range from few tenths of seconds to several seconds) DC bias is applied between the nanoscale tip, and the bottom electrode of the sample, and is swept in a cyclic manner. The resulting external-field caused surface deformation of the sample can be monitored by recording motion of the *z*-piezo (*ΔZ*), which arises to maintain a constant predefined loading force. In a typical AFM system, the fastest response time of the feedback control, and the movement of the *z*-piezo is in the range of few tenth’s of a millisecond (temporal resolution), and therefore is sufficient to monitor the resulting surface displacement/strain caused by slowly varying external fields. Applicability of this approach is limited in the case of thin films to materials with relatively high piezoelectric coefficients as is the case for morphotropic BFO thin films.

1. **Spectroscopic static and dynamic piezoresponse measurements on PbTiO_3_ thin films**

The PbTiO_3_ films of a thickness of approx. 300 nm were grown on SrRuO_3_/SrTiO_3_ (100) substrates by pulsed metal-organic chemical vapor deposition (pulsed-MOCVD) as described in the Refs [[[2]](#endnote-3),[[3]](#endnote-4)]. Figure S2(a) shows the results of static-piezoresponse measurements on the PbTiO_3_ film. Clearly, these measurements reveal butterfly-shaped non-linear hysteretic electromechanical behavior. Local biases (i.e. coercive biases) at which the electrically-induced strain reverses its direction are shown by the intercept (in the range of 5-6 V) of the vertical dotted lines with the horizontal bias axis and corresponds to the reversal of out-of-plane ferroelectric polarization of the PbTiO_3_ film. Further, spectroscopic dynamic piezoresponse measurements (Fig. S2(b)), were performed in the resonant-enhanced PFM mode[[4]](#endnote-5). Local dynamic PFM hysteresis loops were acquired at fixed locations on the film surface as a function of a dc switching bias superimposed on ac modulation bias. As can be seen (Fig. S2(b)), the measured coercive biases (shown by the intercept of dotted vertical lines with the horizontal axis) from the dynamic piezoresponse measurements are in good agreement with the values obtained using the static-piezoresponse approach.

**
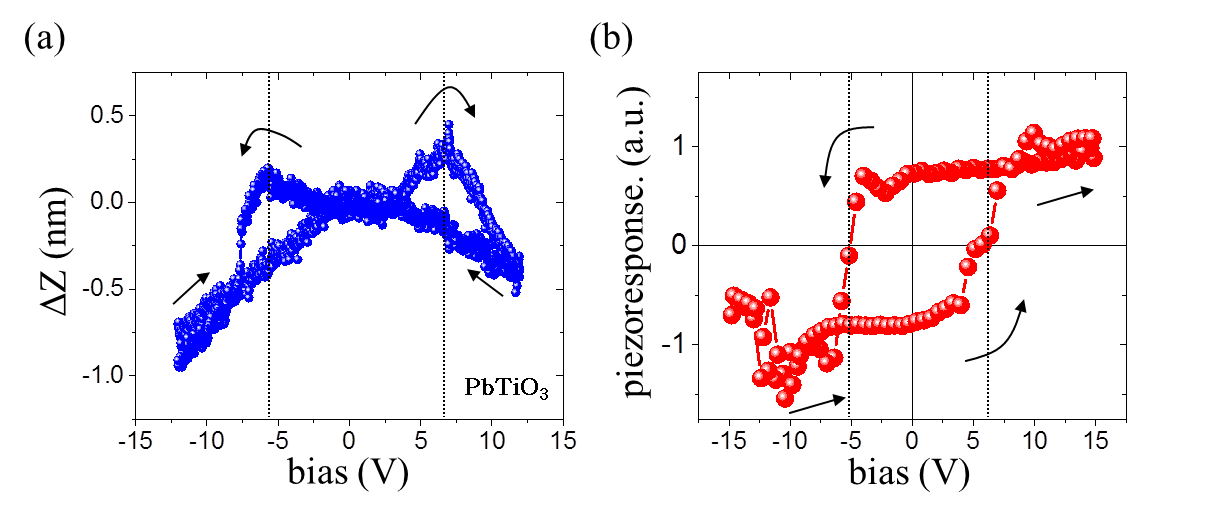
**

**Figure S2. Static and dynamic spectroscopic piezoresponse measurements on a PbTiO_3_ thin film.** (a) Static piezoresponse: spectroscopic local surface displacement as a function of applied external DC bias. (b) Dynamic piezoresponse (PFM) hysteresis loop.

1. **As-grown state of the polarization of the La-doped BFO film**

Figure S3 shows simultaneously acquired topographic and out-of-plane PFM images before and after application of the external electrical bias. In these measurements, the electrical bias was supplied to the bottom electrode, while the conductive AFM tip was grounded. The electrical bias was applied along the dotted lines as shown in Fig. S3(d), and tip scans from left to right. As is clear (Fig. S3), application of +3V along the dotted black line leads to the formation of nanoscale mixed-phase stripes without reversal of the ferroelectric polarization. While application of slightly higher bias (i.e. +5V along the dotted blue line) results in formation of not only nanoscale mixed-phase stripes, but ferroelectric polarization reversal (i.e. polarization pointing up) along the path traversed by the tip. This is elucidated by the newly created line domain, which shows domain walls (zero-PFM amplitude dark lines) in the PFM amplitude image and change in the PFM phase signal by approx. 180° in the PFM phase image. These measurements show that La-doped BFO film in their as-grown state exhibits polarization pointing downwards (i.e. pointing towards the bottom interface).

**
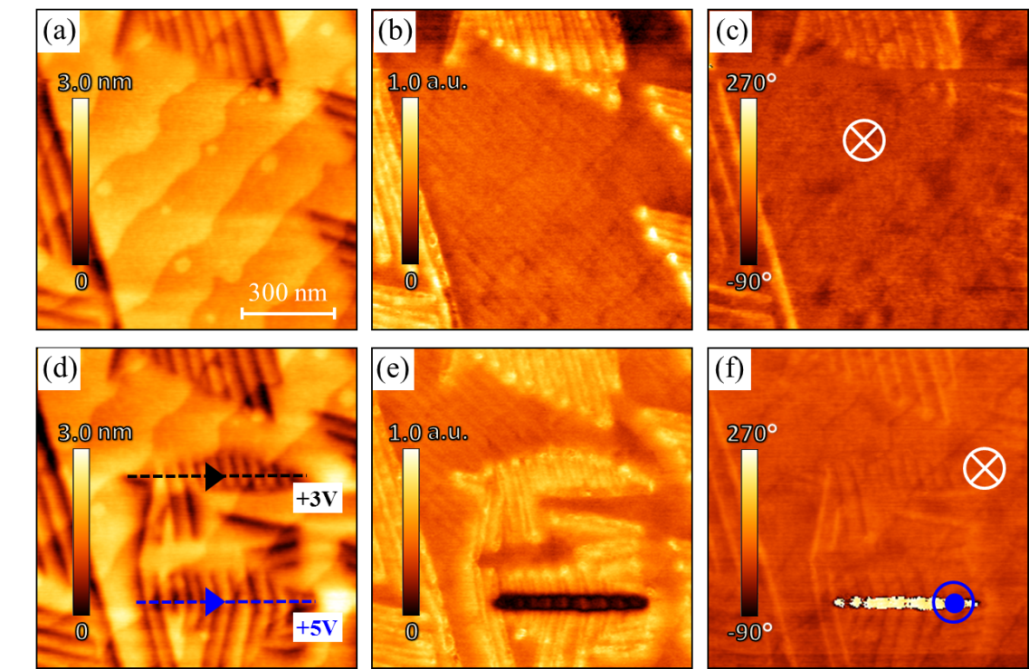
**

**Figure S3. PFM imaging of the La-doped BFO(60nm)/PCMO/LAO film.** (a-c) Topography (a), and the corresponding vertical PFM amplitude (b), and phase (c) images in the as-grown state initially. (d-f) Topography (d), and the corresponding vertical PFM amplitude (e), and phase (f), images of the same area as in (a-c) acquired after application of respective external electrical bias (i.e. +3V along the dotted black line, +5V along the dotted blue line) along the dotted lines as shown in (d). In (d), arrow on the dotted lines indicates the direction of movement of tip (i.e. left to right) during application of the respective electrical biases.

1. **Local spectroscopic dynamic piezoresponse measurements**

Figure S4 shows a typical spectroscopic dynamic piezoresponse hysteresis loop as measured on 60 nm La-doped BFO film using resonant-enhanced PFM mode^4^. The measured local coercive bias values (shown by the intercept of dotted vertical lines with the horizontal axis) are approx. 3 V. However, similar measurements at different spatial locations on the film revealed inhomogeneity in the values of local threshold biases, which were found to be in the range of 3-5 V.

**
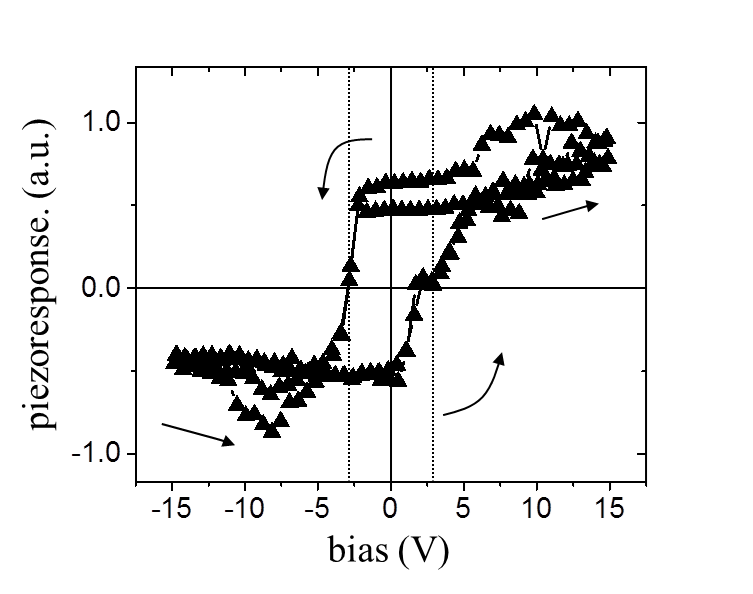
**

**Figure S4.** Dynamic PFM hysteresis loop: local piezoresponse as a function of DC bias acquired on the La-doped BFO film.

1. **Nearly symmetric static piezoresponse measurements for pathway I**

Figure S5 shows static-piezoresponse measurements over a bias range of ± 12V. For the sake of clarity, the initial quarter cycle (see Figs. 1(b-c) in the manuscript) is not shown in the data. As is clear, the acquired response is nearly symmetric, and the magnitude of the threshold biases for reverse structural phase transformations, i.e. from R- to T-like phase transformations are nearly the same for the positive and negative biases. For positive biases the reverse transformation occurs at a bias of approx. +7V (event#3), whereas it occurs at -9V (event#6) for the negative biases. The respective events, i.e. event #3, and 6 in the Figure S5 have been named according to convention developed in the manuscript (see Fig. 1, in the manuscript).

**Figure S5. Static-piezoresponse measurements on La-doped BFO thin film.** Spectroscopic local surface displacement as a function of applied external DC bias.

1. **Phase Evolution analysis- A thermodynamic approach for the epitaxial BFO film**

For a BFO epitaxial film subjected a biaxial misfit strain *u*_m_ from substrate constraint, its free energy can be derived from the standard Gibbs function via the Legendre transform, and given as[[5]](#endnote-6):

with

$$\alpha_{1}^{*}=\alpha_{1}-\frac{(Q_{11}+Q_{12})u_{m}}{S_{11}+S_{12}},$$

$$\alpha_{3}^{*}=\alpha_{1}-\frac{2Q_{12}u_{m}}{S_{11}+S_{12}},$$

$$\alpha_{11}^{*}=\alpha_{11}+\frac{1}{2(S_{11}^{2}-S_{12}^{2})}[S_{11}(Q_{11}^{2}+Q_{12}^{2})-2S_{12}Q_{11}Q_{12}],$$

$$\alpha_{33}^{*}=\alpha_{11}+\frac{Q_{12}^{2}}{S_{11}+S_{12}},$$

$$\alpha_{12}^{*}=\alpha_{12}-\frac{1}{S_{11}^{2}-S_{12}^{2}}[S_{12}(Q_{11}^{2}+Q_{12}^{2})-2S_{11}Q_{11}Q_{12}]+\frac{Q_{44}^{2}}{2S_{44}},$$

$$\alpha_{13}^{*}=\alpha_{12}+\frac{Q_{12}(Q_{11}+Q_{12})}{S_{11}+S_{12}},$$

where $P_{i}$ is polarization component, $E_{i}\begin{matrix} \end{matrix}(i=1,\begin{matrix} \end{matrix}2,\begin{matrix} \end{matrix}3)$ is electric field, $S_{IJ}\begin{matrix} \end{matrix}(I,J=1,\begin{matrix} \end{matrix}2,\begin{matrix} \end{matrix}...,\begin{matrix} \end{matrix}6)$ is elastic compliance constant measured at constant polarization, and $Q_{IJ}$ is the electrostrictive coefficient coupling the elastic and electric field in ferroelectrics, $\alpha_{1}$ and $\alpha_{ij}$ are dielectric constants. All the coefficients used in our calculations are adopted from the Reference [Error: Reference source not found,[[6]](#endnote-7)] and assumed to be independent of temperature, except for$\alpha_{1}=(T-T_{0})/2\varepsilon_{0}C$, where $T_{0}$ and $C$ are the Curie-Weiss temperature and constant.

**References**

1. . Damodaran, A. R. *et al*. Nanoscale structure and mechanism for enhanced electromechanical response of highly strained BiFeO_3_ thin films. *Adv. Mater.* **23**, 3170-3175 (2011). [↑](#endnote-ref-2)
2. 2. Yasui, S. *et al*. Complex domain structure in relaxed PbTiO_3_ thick films grown on (100)_c_SrRuO_3_//(100)SrTiO_3_ substrates. *J. Appl. Phys.* **112**, 052001 (2012). [↑](#endnote-ref-3)
3. . Kamo, T., *et al*. RF magnetron sputtering growth of epitaxial SrRuO_3_ films with high conductivity. *Jpn. J. Appl. Phys.* Part 1 **46**, 6987 (2007). [↑](#endnote-ref-4)
4. . Rodriguez, B. J., Callahan, C., Kalinin, S. V. & Proksch, R. Dual-frequency resonance-tracking atomic force microscopy. *Nanotechnology* **18**, 475504 (2007). [↑](#endnote-ref-5)
5. . Liu, Y. Y., Yang, L. & Li, J. Y. Strain-engineered orthorhombic-rhombohedral phase boundary in epitaxial bismuth ferrite films. *J. Appl. Phys.* **113**, 183524 (2013). [↑](#endnote-ref-6)
6. . Liu, Y. Y. *et al*. Controlling magnetoelectric coupling by nanoscale phase transformation in strain engineered bismuth ferrite. *Nanoscale* **4**, 3175-3183 (2012). [↑](#endnote-ref-7)
